# Nodal modulator (NOMO) is required to sustain endoplasmic reticulum morphology

**DOI:** 10.1016/j.jbc.2021.100937

**Published:** 2021-07-03

**Authors:** Catherine Amaya, Christopher J.F. Cameron, Swapnil C. Devarkar, Sebastian J.H. Seager, Mark B. Gerstein, Yong Xiong, Christian Schlieker

**Affiliations:** 1 Department of Molecular Biophysics and Biochemistry, Yale University, New Haven, Connecticut, USA; 2 Program in Computational Biology and Bioinformatics, Yale University, New Haven, Connecticut, USA; 3 Department of Computer Science, Yale University, New Haven, Connecticut, USA; 4 Department of Statistics and Data Science, Yale University, New Haven, Connecticut, USA; 5 Department of Cell Biology, Yale School of Medicine, New Haven, Connecticut, USA

**Keywords:** endoplasmic reticulum, cell biology, imaging, membrane protein, single-particle analysis, structural model, ATLs, atlastins, Baf A, bafilomycin A, CYT, cytosolic tail, DDM, dodecyl maltoside, EM, electron microscopy, ER, endoplasmic reticulum, Ig, immunoglobulin, LAMP1, lysosomal-associated membrane protein 1, LC3-II, microtubule-associated protein light chain 3, LD, luminal domain, MBP, maltose-binding protein, NCLN, nicalin, NOMO1, nodal modulator 1, PDCs, protein detergent complexes, RTNs, reticulons, SEC-MALS, size-exclusion chromatography linked to multiangle light scattering, TEM, transmission electron microscopy, TM, transmembrane, TMEM147, transmembrane protein 147, UPR, unfolded protein response

## Abstract

The endoplasmic reticulum (ER) is a membrane-bound organelle responsible for protein folding, lipid synthesis, and calcium homeostasis. Maintenance of ER structural integrity is crucial for proper function, but much remains to be learned about the molecular players involved. To identify proteins that support the structure of the ER, we performed a proteomic screen and identified nodal modulator (NOMO), a widely conserved type I transmembrane protein of unknown function, with three nearly identical orthologs specified in the human genome. We found that overexpression of NOMO1 imposes a sheet morphology on the ER, whereas depletion of NOMO1 and its orthologs causes a collapse of ER morphology concomitant with the formation of membrane-delineated holes in the ER network positive for the lysosomal marker lysosomal-associated protein 1. In addition, the levels of key players of autophagy including microtubule-associated protein light chain 3 and autophagy cargo receptor p62/sequestosome 1 strongly increase upon NOMO depletion. *In vitro* reconstitution of NOMO1 revealed a “beads on a string” structure likely representing consecutive immunoglobulin-like domains. Extending NOMO1 by insertion of additional immunoglobulin folds results in a correlative increase in the ER intermembrane distance. Based on these observations and a genetic epistasis analysis including the known ER-shaping proteins Atlastin2 and Climp63, we propose a role for NOMO1 in the functional network of ER-shaping proteins.

As the largest, single-membrane–bound organelle, the endoplasmic reticulum (ER) is responsible for critical and diverse functions, including lipid synthesis, folding and export of membrane and secretory proteins, and calcium storage (1, 2, 3). These responsibilities are divided into three structurally distinct regions, namely the nuclear envelope, sheets, and tubules (4). These regions partition protein synthesis and folding to the sheets, and organelle fission and calcium storage to tubules (5). The structural integrity of these regions is maintained and regulated by unique membrane-shaping proteins.

The membrane-shaping proteins necessary to support the curvature of ER tubules have largely been established (6), which include reticulons (RTNs), atlastins (ATLs), and receptor expression-enhancing proteins (7, 8). The prominent structural motif shared by these proteins is a transmembrane (TM) hairpin, which serves as a wedge that is inserted into the outer lipid layer of the ER membrane to impose high curvature on the membrane and help create a tubular shape. In addition, ATLs have a cytosolic GTPase domain responsible for fusion and tethering of tubules and creating the connected reticular network of the ER (8). Depletion of ATLs results in ER tubules becoming abnormally long and unbranched and disrupts ER tubule functionality (9, 10). This disruption demonstrates the critical role of maintaining ER membrane morphology for the function of the ER. The importance of understanding how the ER maintains structural integrity is highlighted by diseases that occur when the functions of ER-shaping proteins are disrupted. Mutations in tubule-shaping proteins, such as in ATLs, spastin, RTNs, and receptor expression-enhancing protein 1, are associated with diseases such as amyotrophic lateral sclerosis, hereditary spastic paraplegia, and other neurodegenerative disorders (11, 12, 13).

Although tubule-shaping proteins have been well established, much remains to be learned about sheet morphology. The maintenance of sheet spacing is largely attributed to a highly abundant protein, Climp63, an ER resident microtubule-binding protein that features a long coiled-coil domain in the ER lumen (14, 15, 16), whereas the high curvature edges of the sheets are stabilized by tubule-shaping proteins such as RTNs (7, 17, 18). Initially, it was proposed that the coiled-coil domain of Climp63 dimerizes across the ER lumen to support an intermembrane distance of about 60 nm (16). Indeed, modulating the length of the Climp63 coiled-coil domain was shown to correlatively affect the ER luminal distance (19). Kinectin and p180 have also been proposed to contribute to the flatness of sheets. Despite these contributions to maintaining sheet morphology, simultaneous depletion of Kinectin, p180, and Climp63 or Climp63 alone does not result in a loss of sheets. Rather, the ER diameter is uniformly decreased to 30 nm (16, 19). Climp63 has also been proposed to keep the opposing sheet membranes from collapsing into each other (20). However, Climp63 depletion does not lead to a loss of sheets, and no functional perturbations of the ER have been reported. These observations suggest that additional, yet unidentified, sheet-shaping proteins exist to prevent disruption to ER sheet functions.

Here, we use a proximity ligation-based approach to identify additional ER-luminal proteins that could contribute to membrane spacing. We identified nodal modulator 1 (NOMO1), a widely conserved type1 TM glycoprotein, as an abundant luminal constituent of the ER. Depletion of NOMO1 in a tissue culture model perturbs ER morphology, while its overexpression imposes a defined intermembrane spacing on the ER. Furthermore, *in vitro* reconstitution and low-resolution electron microscopy (EM) collectively suggest that NOMO1 is a rod-shaped molecule, featuring immunoglobulin (Ig) folds that are arranged as “pearls on a string”. Based on these observations, as well as a genetic epistasis analysis including several ER-shaping proteins, we place NOMO1 in a functional network of proteins responsible for establishing and maintaining the morphology of the ER.

## Results

### Identification of NOMO1 as an abundant, ER-luminal protein

**Figure 1:**
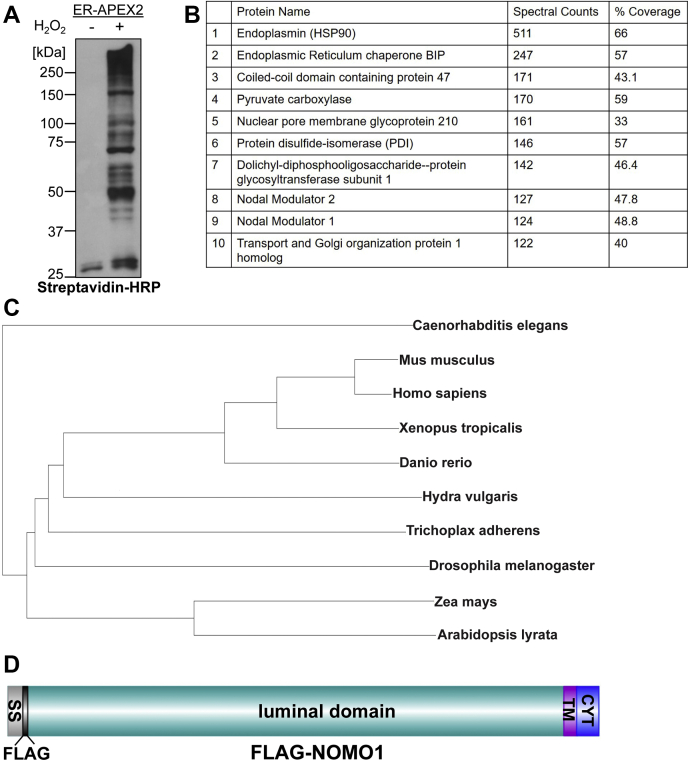
**Identification of NOMO1 as an abundant and conserved ER-resident protein.***A*, cells expressing ER-APEX2 were treated with biotin-phenol in absence or presence of hydrogen peroxide, lysed, and subjected to Western blotting using streptavidin-HRP. *B*, table of top-10 most abundant proteins from MS analysis in order of spectral count; % coverage is the sequence coverage of the protein based on the peptide sequences identified. *C*, phylogenetic tree of NOMO1 homologs in indicated metazoan and plant species. *D*, FLAG-NOMO1 domain structure. Note that the FLAG tag was inserted between the cleavable signal sequence (SS) and the luminal domain. CYT, cytosolic tail; ER, endoplasmic reticulum; NOMO1, nodal modulator 1; TM, transmembrane domain.

To identify potential sheet-shaping proteins, we used a proximity ligation approach. Previous proteomes of the ER were obtained by subcellular fractionation–based techniques that encompassed the entire ER membrane network (21, 22), whereas we were specifically interested in the ER lumen. To this end, we used an engineered monomeric peroxidase (APEX2) (23). In the presence of hydrogen peroxide, APEX2 creates biotin-phenoxyl radicals that will biotinylate proteins in a 20-nm radius (24, 25, 26). We used ER-APEX2, a construct previously shown to specifically localize to the ER lumen by virtue of a signal sequence (27). This construct was expressed in HeLa cells that were then incubated with biotin and treated with hydrogen peroxide to conjugate biotin to ER luminal proteins. The control sample was transfected with ER-APEX2, but no hydrogen peroxide was added. Because robust, hydrogen peroxide–dependent labeling was observed for a variety of proteins (Fig. 1*A*), we performed an analogous experiment on a larger scale and analyzed the resulting eluates *via* MS after tryptic digestion. As expected, the most abundant species identified included constituents of ER protein synthesis and folding machinery (Fig. 1*B*), including the ER chaperones BiP, protein disulfide isomerase, endoplasmin, and CCD47, all of which are known residents of the ER lumen (28, 29). In addition, NOMO2 and NOMO1 were the eighth and ninth most abundant proteins, respectively, identified as judged by spectral counts, with high sequence coverage (48%) (Fig. 1*B*, Table S1).

NOMO1 is a type I TM protein that is conserved across all metazoans (30). Notably, NOMO homologs are also present in plants, both in monocotyledons (*Zea mays*) and dicotyledons (*Arabidopsis lyrata*) (Fig. 1*C*). While other metazoan organisms specify a single copy of NOMO, three copies of NOMO are present in the human genome designated: *NOMO1*, *NOMO2*, and *NOMO3* (31). NOMO1 and NOMO2 specify a 134-kDa membrane protein composed of an N-terminal 1124-residue luminal domain (LD), a TM domain, and a short, 40-residue cytosolic tail (CYT) domain. The LDs of the three proteins are identical except for six amino acids (Fig. S1*A*). NOMO2 has a CYT domain that is 45 residues longer than NOMO1 and NOMO3, resulting in a 139-kDa membrane protein. This extremely high similarity suggests that NOMO orthologs have arisen from recent gene duplication events and have identical or similar cellular functions.

To begin to understand which function NOMO might have in the ER, we used BLAST searches, secondary structure predictions, and fold recognition programs to identify homology to proteins of known structure. Although these searches did not reveal related human proteins, NOMO1 is predicted to form a beta sheet–rich structure (Fig. S1*B*) by using Phyre2 (32). Consistently, a significant structural degree of similarity was detected between NOMO1 and several bacterial Ig fold proteins. The highest similarity was observed for BaTIE, a sortase-anchored surface protein from *Bacillus anthracis* (33), featuring four tandem Ig domains of 19-nm length. This predicted structural homology led us to hypothesize that NOMO1 might adopt an extended rod structure that could serve as a structural component to support membrane spacing.

### NOMO depletion results in altered ER morphology

**Figure 2:**
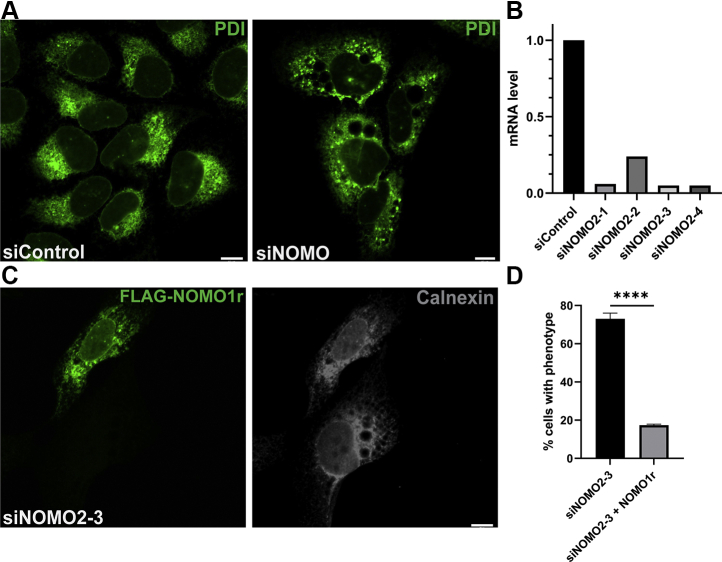
**NOMO depletion results in profound changes of ER morphology.***A*, confocal images of U2OS cells transfected with the respective siRNA for 48 h. Protein disulfide isomerase (PDI) is used as the ER marker. *B*, quantification of mRNA level of each NOMO siRNA by qPCR. *C*, representative image of the phenotypic rescue of the NOMO knockdown phenotype by an siRNA-resistant construct, FLAG-NOMO1r. Calnexin is used as the ER marker. *D*, quantification of rescuing ability of FLAG-NOMO1r, n = 100, N = 3, *p* < 0.05. The *asterisks* denote *p* < 0.005 compared with the control. Error bars indicate SD. All scale bars are 10 μm. ER, endoplasmic reticulum; NOMO1, nodal modulator 1.

As a first test to determine if NOMO depletion contributes to ER morphology, we depleted NOMO in U2OS cells using siRNA. Owing to the high genomic similarity between *NOMO1*, *NOMO2*, and *NOMO3*, siNOMO1 targets all three corresponding mRNAs. In the following text, we will refer to the experimental condition simultaneously depleting NOMO1, NOMO2, and NOMO3 as NOMO. The canonical nomenclature of NOMO1 will be used for experiments based on the specific NOMO1 cDNA or protein. NOMO depletion caused a striking rearrangement of the ER network, and large holes in the ER of up to 5-μm diameter were visible by using immunofluorescence microscopy (Fig. 2*A*). Attempts at generating a CRISPR/Cas9 NOMO KO cell line were unsuccessful. Although single cell colonies were obtained in which the hole phenotype was visible, cells were not viable in culture after several passages, suggesting an important, if not essential, function.

To demonstrate that the siRNA-induced phenotype was specifically due to NOMO depletion, a NOMO1 rescue construct, FLAG-NOMO1r, was designed by introducing silent mutations into the targeting site of siRNA #3. This siRNA depleted NOMO mRNA by over 90% as quantified by qPCR (Fig. 2*B*). FLAG-NOMO1r reproducibly reduced the ER phenotype from 68% penetrance to 20%, providing further evidence that the hole phenotype observed is specifically caused by NOMO depletion (Fig. 2, *C* and *D*). Because the simultaneous depletion of all three NOMO orthologs can be rescued by FLAG-NOMO1r alone, we conclude that NOMO1 has a function in the context of ER morphology.

### Genetic interactions between NOMO and known ER-shaping proteins

**Figure 3:**
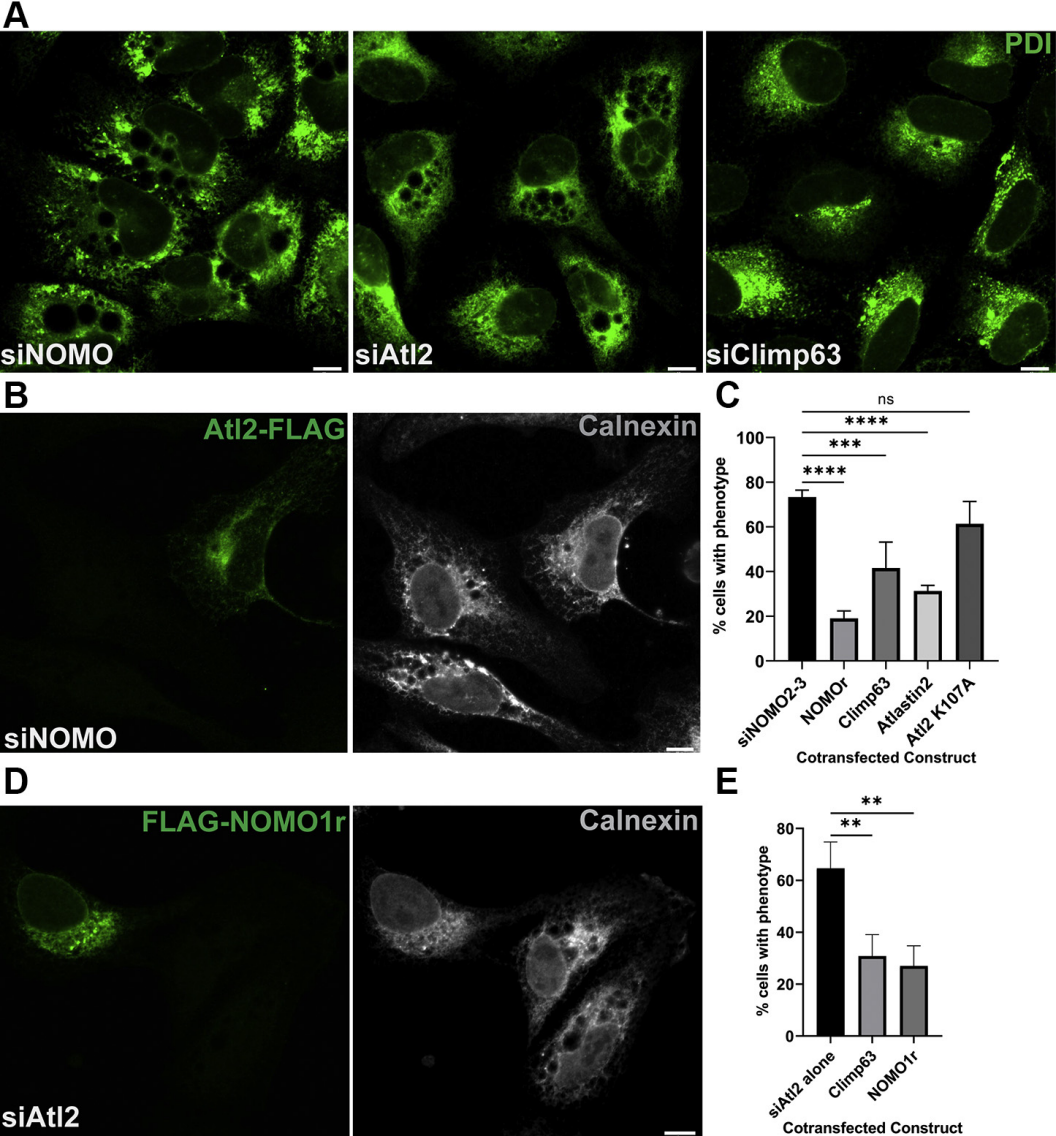
**Epistasis analysis of known ER-shaping proteins and NOMO1.***A*, U2OS cells were treated with respective siRNA for 48 h and stained with PDI as an ER marker. *B*, representative image of Atl2-FLAG overexpression (*left panel*) rescuing the NOMO KD phenotype as judged by PDI staining (*right panel*). *C*, quantification of the ability of ER-shaping proteins to rescue the NOMO KD ER phenotype, n = 100, N = 4. Error bars indicate SD. ∗∗∗*p* < 0.002; ∗∗∗∗*p* < 0.0001. *D*, representative image of FLAG-NOMO1r overexpression (*left panel*) rescuing Atl2 KD phenotype (*right panel*). *E*, quantification of NOMO1 and Climp63 overexpression rescuing the Atl2 KD phenotype, n = 100, N = 3. Error bars indicate SD. ∗∗*p* < 0.01. Scale bars are 10 μm. ER, endoplasmic reticulum; NOMO1, nodal modulator 1; ns, not significant; PDI, protein disulfide isomerase.

From a topological perspective, the predicted domain architecture of NOMO is reminiscent of the structural domain composition of Climp63 that includes a sizeable LD expanding into the ER lumen, a TM domain, and a short CYT (15). Therefore, we sought to compare whether Climp63 depletion caused similar defects in ER morphology as NOMO depletion. Depletion of Atl2 was included as a tubule-shaping protein for comparison. Surprisingly, Atl2 depletion resulted in similar holes as those caused by NOMO depletion, whereas Climp63 depletion had no effect on ER morphology when visualized by immunofluorescence microscopy (Fig. 3*A*).

Next, we asked if NOMO exhibits epistatic relationships with Atl2 or Climp63. First, we tested whether the overexpression of these known ER-shaping proteins modulates the observed hole phenotype. We transfected Atl2-FLAG into NOMO-depleted cells and observed that Atl2-FLAG overexpression could significantly rescue the NOMO knockdown phenotype (Fig. 3, *B* and *C*). Because Atl2 is required for ER fusion, we hypothesized that the fusogenic activity is required for this effect. To this end, a rescue assay was performed with a GTPase mutant of Atl2 that cannot fuse ER membranes, Atl2 K107A (34). This Atl2 mutant did not rescue the NOMO knockdown hole phenotype (Fig. 3*C*), indicating that the rescue ability of Atl2 relies on the fusogenic activity. Furthermore, in an analogous experiment, we found that Climp63-FLAG did rescue the hole phenotype under NOMO depletion to a similar extent compared with Atl2 (Fig. 3*C* and Fig. S2*A*).

Finally, because Atl2 depletion results in a similar hole phenotype, we performed the reciprocal rescue assays of cotransfecting NOMO1-FLAG or Climp63-FLAG into Atl2-depleted cells. We found that NOMO-FLAG and Climp63-FLAG both significantly reduced the penetrance of the Atl2-depletion phenotype (Fig. 3, *D* and *E* and Fig. S2*B*). In conclusion, the observed genetic interactions among NOMO1, Climp63, and Atl2 are consistent with the interpretation that NOMO contributes to the elaborate network of ER-shaping proteins.

### Ultrastructural and compositional characterization of hole phenotype

**Figure 4:**
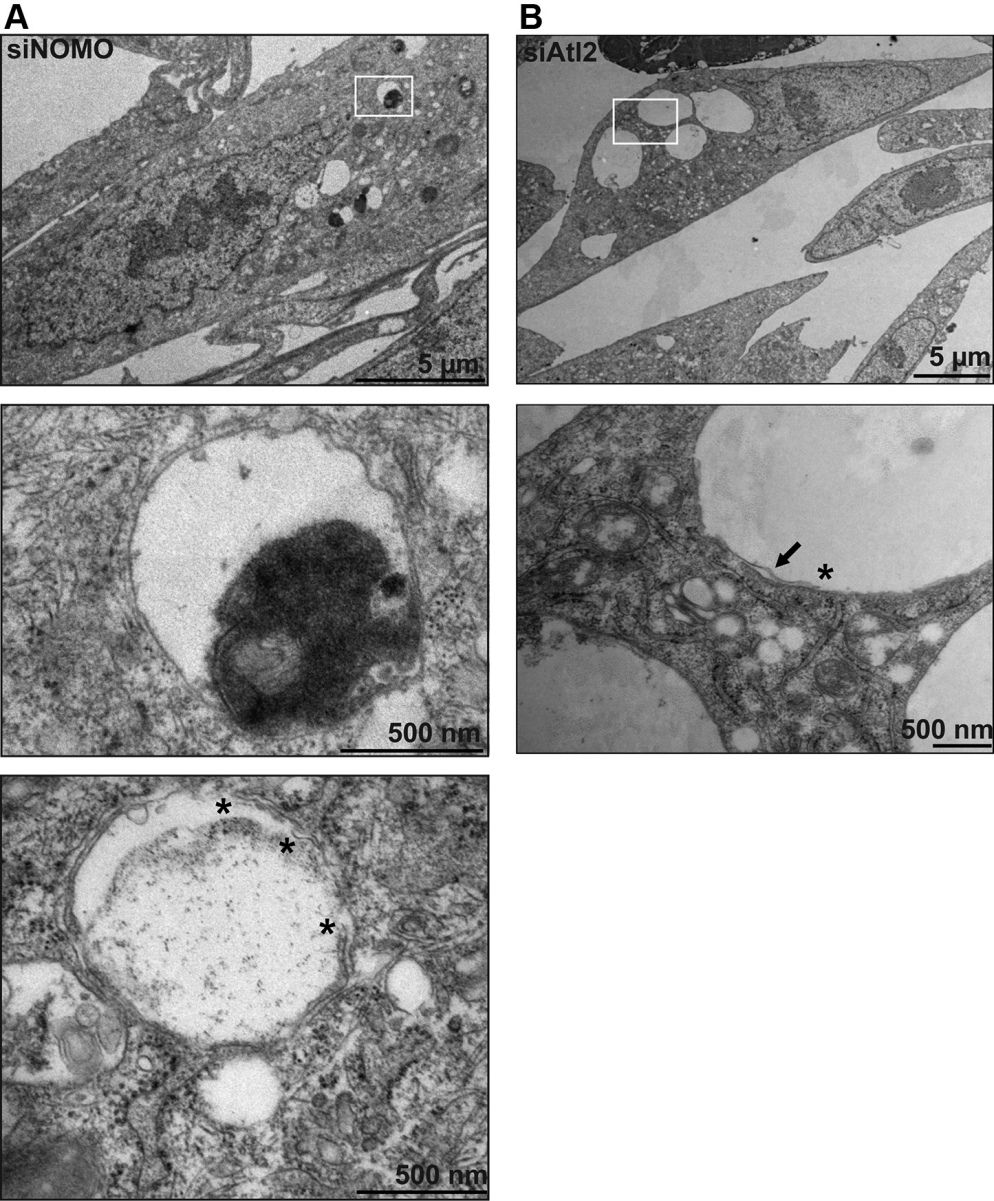
**EM analysis of NOMO and Atl2-depleted cells.***A*, U2OS cells were successively treated with two doses of siNOMO 24 h apart and fixed 48 h after the second dose for EM processing. The *white square* in the *top panel* identifies selection for the *middle panel*. *Asterisks* in *bottom panel* denote free membrane ends. *B*, U2OS cells successively treated with two doses of siAtl2 as described in panel *A*. The *arrow* in *second panel* indicates an identified membrane outlining the hole. EM, electron microscopy; NOMO, nodal modulator.

To further explore the relationship between holes and the ER membrane, we processed U2OS cells depleted of NOMO for transmission electron microscopy (TEM). The holes often appeared to be devoid of any internal electron density and were delineated by membranes in various instances. In general, we encountered fixation issues resulting in suboptimal preservation of holes, possibly because of their large size and low interior content. While these fixation issues generally complicated direct visualization of membrane continuity, we observed in several cases that multiple membranes surrounded one hole (Fig. 4*A*, bottom panel). For comparison, we performed TEM analysis of U2OS cells under Atl2 depletion and observed similar membrane-delineated holes (Fig. 4*B*). These results support the idea that a similar net result is obtained in response to the depletion of either NOMO or Atl2. Finally, we noted electron-dense structures adjacent to or inside a subset of the holes under NOMO depletion (Fig. 4*A*, top and middle panels). While our attempts to obtain three-dimensional information for these ER fenestrations *via* EM tomography failed because of difficulties with structure preservation during fixation, it seems reasonable to speculate that the holes correspond to spherical, membrane-delineated objects.

**Figure 5:**
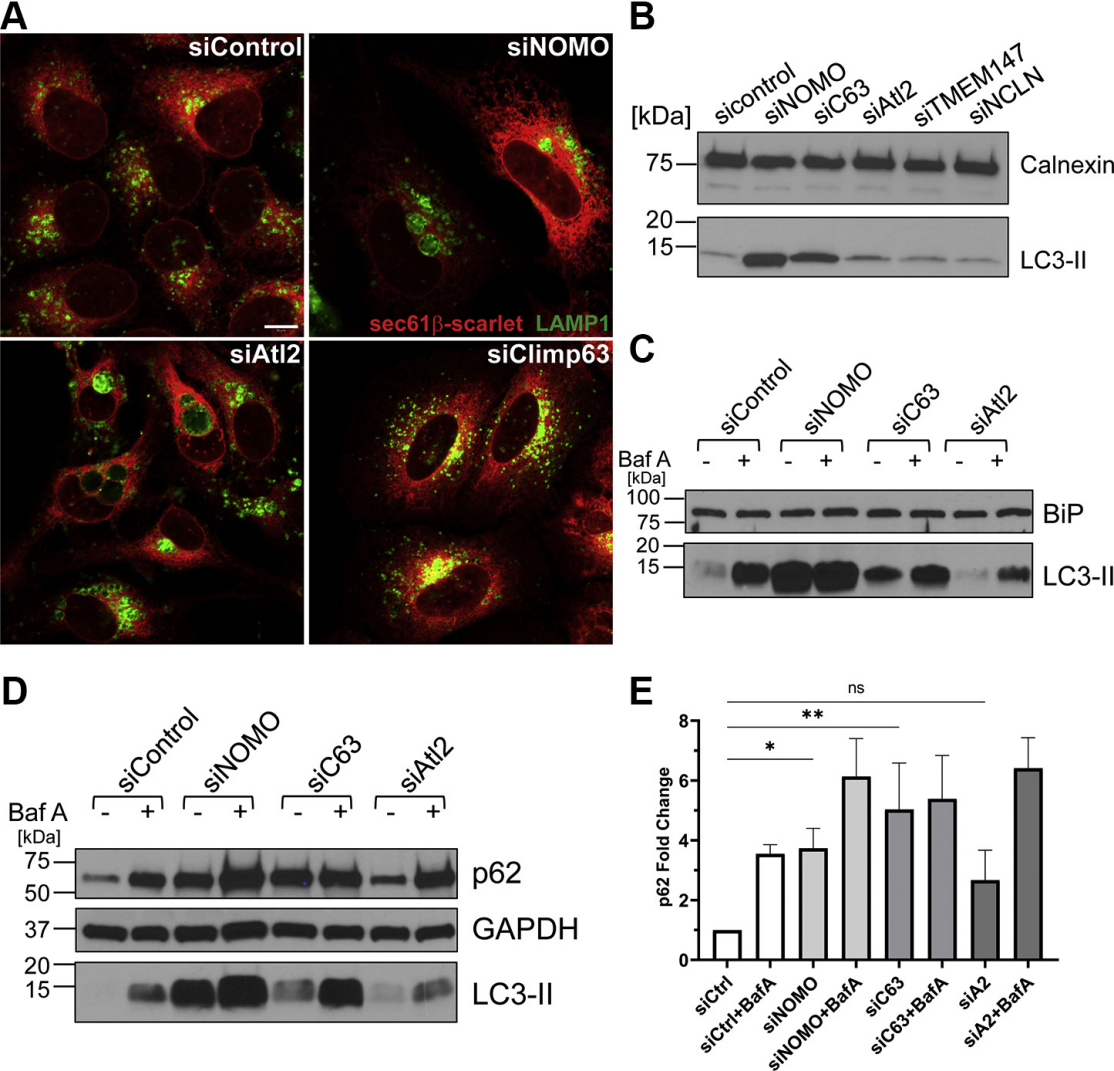
**Sheet disruption increases autophagy.***A*, representative images of U2OS cells treated with the respective siRNA to identify lysosome localization using LAMP1 as a marker. The scale bar is 10 μm. *B*, immunoblot with calnexin and LC3 antibodies using U2OS cells extracts treated with the indicated siRNA. *C*, immunoblot with BiP and LC3 antibodies of the Baf A-treated samples under the respective siRNA conditions. *D*, immunoblot using p62, GAPDH, and LC3 antibodies of the Baf A-treated samples under the respective siRNA conditions. *E*, quantification of the fold change in p62 levels calculated by dividing p62 intensity by GAPDH intensity and normalizing to the untreated control sample. ∗*p* < 0.05; ∗∗*p* < 0.01. Baf A, bafilomycin A; LAMP1, lysosomal-associated protein 1.

To determine if these electron-dense structures represent lysosomal compartments, U2OS cells were treated with siNOMO, siAtl2, or siClimp63 and analyzed by immunofluorescence microscopy using a lysosomal-associated protein 1 (LAMP1)-specific antibody. Indeed, we observed a large accumulation of LAMP1 signal in the ER holes resulting from NOMO and Atl2 depletion (Fig. 5*A*). The observed increase in lysosome size and accumulation compared with control cells could be an indicator of increased autophagy (35). To address this point, we monitored LC3 processing by immunoblotting. We observed an increase of microtubule-associated protein light chain 3 (LC3-II) under NOMO depletion compared with control, which is indicative of autophagy induction or dysregulation, while LC3-I was mostly at or below the detection limit in the short exposures that were necessary to record the higher LC3-II levels (Fig. 5, *B*–*D*). We also monitored LC3 levels under the depletion of Climp63 and Atl2, and NOMO binding partners transmembrane protein 147 (TMEM147) and nicalin (NCLN) (36, 37). An increase in LC3-II was also observed upon Climp63 depletion, although less pronounced compared with NOMO depletion (Fig. 5*B*), whereas the other tested conditions did not significantly increase LC3-II levels.

Next, we treated cells with bafilomycin A (Baf A) to inhibit autophagy (38), which causes an increase in LC3-II levels. Although siCtrl, siClimp63, and siAtl2 resulted in an increase in LC3-II levels upon Baf A addition compared with the control siRNA, siNOMO LC3-II levels were close to saturation levels even without Baf A treatment (Fig. 5*C*). We did not observe an increase in BiP levels under NOMO depletion, which would have indicated an induction of the unfolded protein responses (UPRs) due to ER stress (Fig. 5*C*) (39). Apart from LC3, accumulation of the autophagy receptor p62 is a commonly used readout in the context of autophagy (38, 40). We therefore compared p62 levels in control cells with those silenced for Nomo1, Climp63, or Atl2, both in the absence and presence of Baf A treatment. NOMO, Climp63, and Atl2 depletions resulted in increased p62 levels, with Atl2 depletion causing the smallest effect. When quantified, NOMO-depleted cells not treated with Baf A had p62 levels similar to the control sample treated with Baf A (Fig. 5, *D* and *E*). However, we still observed an increase in p62 levels in NOMO-depleted cells upon addition of Baf A. Similar results were obtained upon depletion of Climp63, although the additive effect of Baf A treatment was less pronounced for p62 levels compared with NOMO depletion (Fig. 5, *D* and *E*). Regardless, to our knowledge, this is the first indication that Climp63 depletion can materially perturb a cellular function. Our results additionally suggest that the functional relationship of membrane-shaping proteins and the autophagic/lysosomal route warrants closer scrutiny in the future.

### NOMO overexpression imposes ER sheet morphology

**Figure 6:**
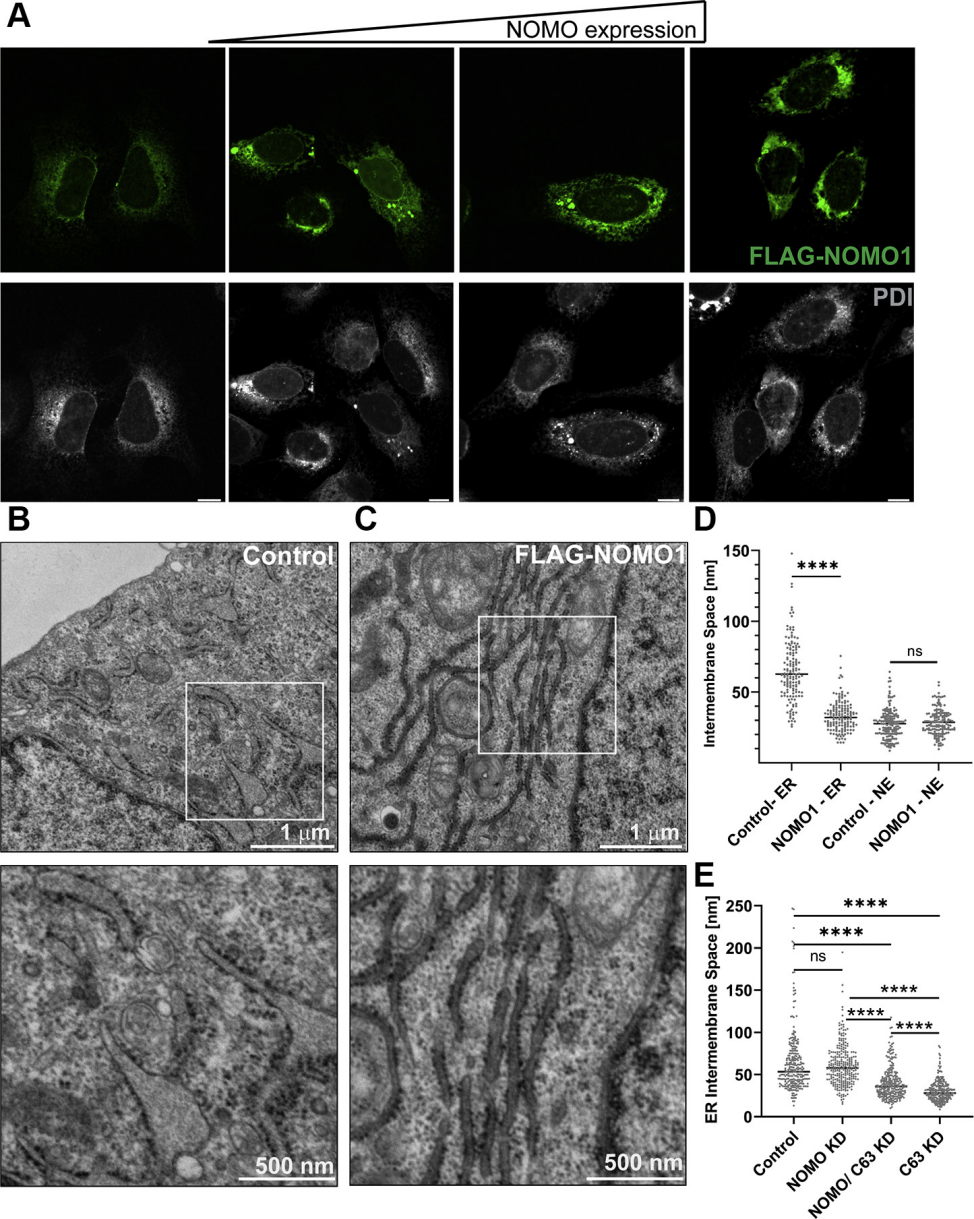
**NOMO1 restricts the lumen of the ER.***A*, increasing expression of FLAG-NOMO1 in U2OS cells using PDI as the ER marker. All scale bars are 10 μm. *B*, HeLa cells transfected with the empty vector pcDNA3 as a control. The *white box* in the *top panel* identifies selected zoomed ER membrane area in the *bottom panel*. *C*, HeLa cells transfected with FLAG-NOMO1. The *white box* in *top panel* identifies selected zoomed ER membrane area in *bottom panel*. *D*, intermembrane space of HeLa cell ER and NE from panels *B* and *C*. *Asterisks* indicate *p* < 0.0001. *E*, intermembrane space of ER and NE cross-sections of U2OS cells treated with the respective siRNAs. *Asterisks* indicate *p* < 0.0001. ER, endoplasmic reticulum; NE, nuclear envelope; NOMO1, nodal modulator 1; ns, not significant; PDI, protein disulfide isomerase.

We hypothesized that if NOMO contributes to ER intermembrane spacing similar to Climp63, then overexpressing NOMO1 should affect the spacing of the ER lumen as well as leading to an increased sheet morphology of the ER (19). To this end, we overexpressed FLAG-NOMO1 in U2OS cells and subjected the cells to fixation and confocal microscopy. Representative confocal images show that at low- to moderate-expression levels, FLAG-NOMO1–transfected cells feature essentially normal ER morphology (Fig. 6*A*). In cells with higher FLAG-NOMO1 expression levels (Fig. 6*A*, rightmost panel), we observed an increase in continual ER areas reminiscent of ER sheets when compared with untransfected cells. (Fig. 6*A*). To determine if ER sheet spacing was indeed affected, we subjected HeLa cells overexpressing FLAG-NOMO1, as well as control cells transfected with an empty vector, to TEM imaging. Cells overexpressing FLAG-NOMO1 had a constricted ER lumen diameter compared with the control cells (Fig. 6, *C* and *D*). When quantified, FLAG-NOMO1 overexpression reduced the ER lumen from an average intermembrane distance of 66 nm to 33 nm (Fig. 6*E*). Interestingly, a similar reduction in the ER lumen diameter results from depleting Climp63, where the ER lumen is decreased to an intermembrane space of 30 nm (16), potentially suggesting that NOMO might contribute to maintaining this smaller diameter of 30 nm.

We next asked whether NOMO and Climp63 depletion, either alone or in combination, results in changes in membrane spacing. Would the luminal space become wider than in a WT cell? The diameter could alternatively decrease as sheet-shaping proteins have been proposed to help keep the opposing sheet membranes from collapsing into each other (20). NOMO depletion caused a small, however, not significant increase in the intermembrane distance, whereas Climp63 depletion resulted in a significant reduction in membrane spacing, as was previously reported (Fig. 6*E*) (16, 19). Depletion of NOMO1 in Climp63-silenced cells led to a small, yet statistically significant, increase in membrane spacing relative to a Climp63 knockdown (Fig. 6*E*). Thus, the ER intermembrane distance can be modulated both by overexpression and depletion of NOMO1, with overexpression causing more pronounced effects than depletion.

### NOMO is a rod-shaped molecule

**Figure 7:**
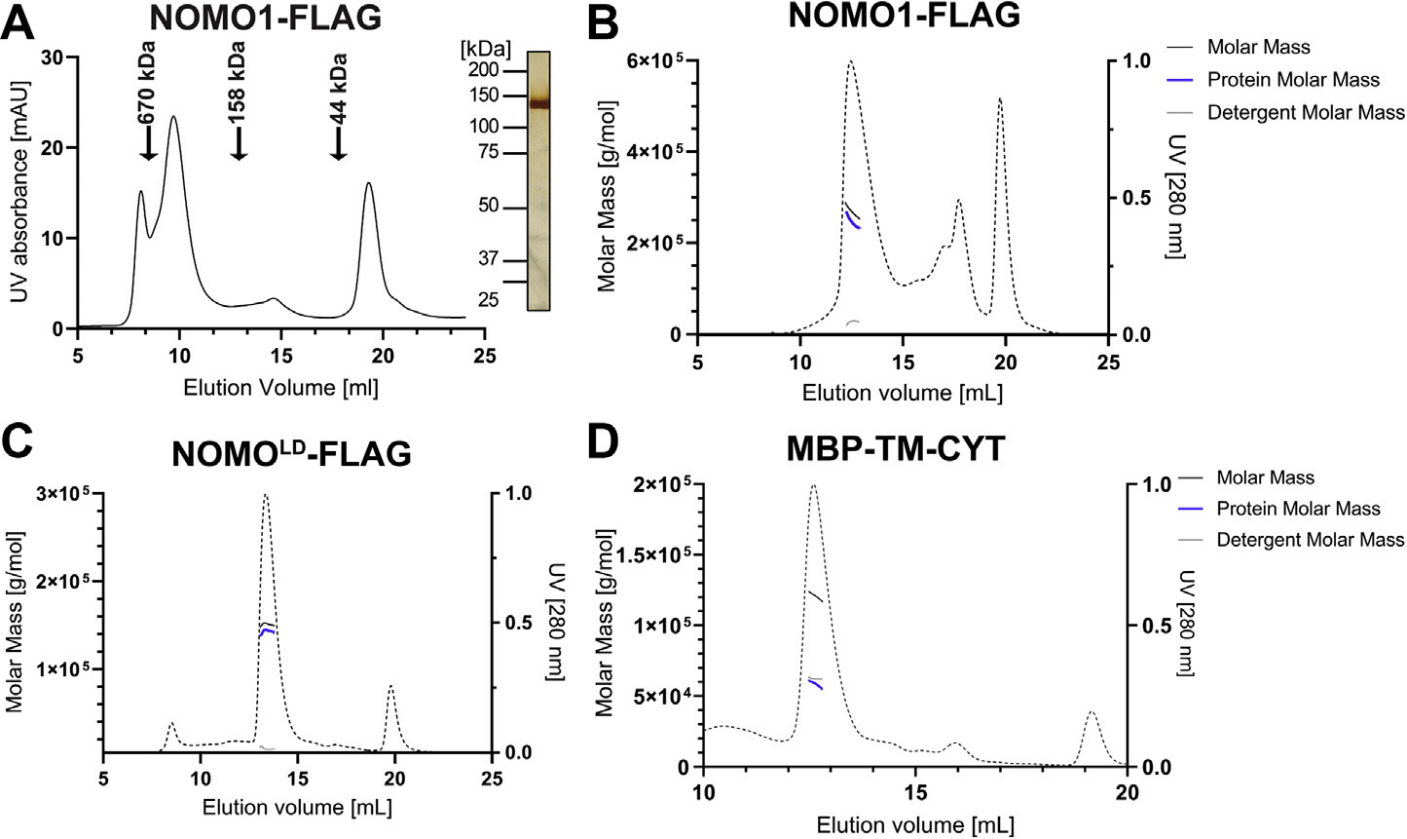
**Determination of the NOMO1 oligomeric state.***A*, elution profile of NOMO1-FLAG on a S200 column. *Insert*, SDS-PAGE/silver stain of NOMO1-FLAG fraction obtained from preparative SEC. *B*, in-line SEC-MALS profile of NOMO1-FLAG subjected to a Superose 6 column. The *dashed line* is the UV trace, molar mass represents the total molar mass of the PDC, protein molar mass is the corrected molar mass to remove contribution of detergent. *C*, SEC-MALS profile of NOMO^LD^-FLAG subjected to a Superose 6 column; lines as defined in panel *B*. *D*, in-line SEC-MALS analysis of MBP-TM-CYT subjected to a S200 Increase column. CYT, cytosolic tail; NOMO1, nodal modulator 1; SEC-MALS, size-exclusion chromatography linked to multiangle light scattering; TM, transmembrane.

Because the overexpression of NOMO causes a uniform restriction of ER intermembrane spacing, we hypothesized that NOMO may support sheet structure by dimerizing across the sheet membranes to support the luminal diameter as originally proposed for Climp63 (16). To determine if NOMO could oligomerize, NOMO1-FLAG (135 kDa) was purified from Expi293F cells in the presence of the mild detergent dodecyl maltoside (DDM) and analyzed by size-exclusion chromatography (SEC) (Fig. 7*A*). NOMO1-FLAG eluted at an apparent mass of about 500 kDa based on the elution position, which would correspond to a tetramer of NOMO1. However, the potentially elongated form and the correspondingly large apparent stokes radius of NOMO1 could be contributing to significant experimental error, as well as the detergent micelle that is presumably associated with the TM.

To more accurately determine the oligomeric state and molecular mass of NOMO1-FLAG, we coupled SEC to multiangle light scattering (SEC-MALS). The SEC-MALS analysis revealed a radius of gyration of about 15 nm (Fig. 7*B*) corresponding to the main peak in the elution profile. Reconstitution of membrane proteins using detergents often leads to the formation of protein detergent complexes (PDCs), and therefore, we performed a protein conjugate analysis on this peak to delineate the molar mass of the protein component from the total molar mass of the PDC. According to this analysis, the molar mass of NOMO1 ranged from ∼270 kDa on the left side of the peak to ∼230 kDa toward the right side of the peak, with each value representing the mass after correction for detergent contribution (Fig. 7*B*). This apparent polydispersity across the peak is not uncommon for oligomeric membrane proteins reconstituted in the presence of detergents (41, 42, 43). Based on the observed mass range, we suggest that NOMO1 forms a low-affinity dimer; however, additional equilibrium methods will be required to definitively determine the oligomeric state.

To avoid complications arising from the detergent micelle, we also performed SEC-MALS analysis with a NOMO^LD^-FLAG construct (128 kDa) lacking both the TM domain and the CYT domains. After a protein conjugate analysis, the monodisperse peak correlated to a mass of 142 kDa, consistent with NOMO^LD^-FLAG being a monomer (Fig. 7*C*). Furthermore, NOMO^LD^-FLAG had a similar radius of gyration (∼14 nm) as full-length NOMO1-FLAG. These data argue in favor of full-length NOMO forming a potential parallel dimer, but as mentioned before, additional experiments will be required to rigorously test this proposal in the future.

Given that the LD was not sufficient for oligomerization, we tested if NOMO could dimerize *via* the TM and CYT domains. To this end, the TM-CYT domains were fused to maltose-binding protein (MBP) to yield 2xFLAG-MBP-TM-CYT, which was expressed, purified from Expi293F cells, and subjected to SEC-MALS analysis. The molar mass of the MBP-TM-CYT peak was estimated to be ∼120 kDa from SEC-MALS. However, protein conjugate analysis revealed that the molar mass of MBP-TM-CYT is 58 kDa and the detergent contributed 62 kDa to the apparent molar mass of the PDC (Fig. 7*D*), revealing that MBP-TM-CYT is a monomer. At this point, we cannot rule out that MBP sterically interferes with a possible dimerization or that several distinct structural features in NOMO1 contribute to dimerization collectively but are insufficient to confer dimerization individually.

### NOMO1 adopts a “beads on a string” morphology

**Figure 8:**
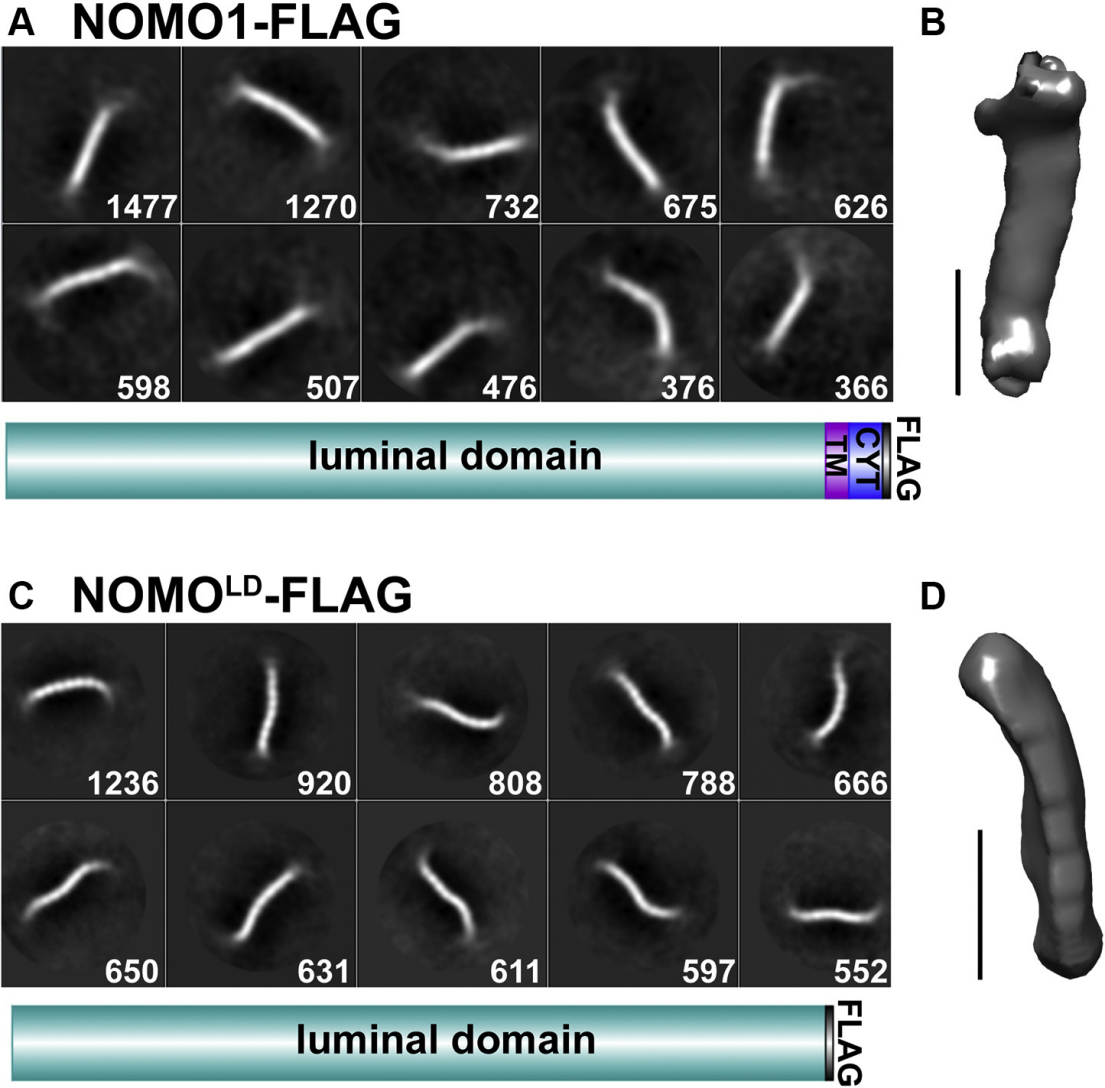
**Single-particle analysis of NOMO1.***A*, top-10 two-dimensional class averages of ∼7000 picked negative-stain NOMO1-FLAG particles, numbers of particles per class in *squares*. Mask diameter is 40 nm. A construct layout is inserted to clarify protein domains. *B*, three-dimensional reconstruction from panel *A*; the scale bar is 10 nm. *C*, top-10 two-dimensional class averages of ∼10,000 picked negative-stain NOMO^LD^-FLAG particles, the number of particles per class in *squares*. Mask diameter is 30 nm. A construct layout is inserted. *D*, three-dimensional reconstruction from panel *C*, the scale bar is 10 nm. CYT, cytosolic tail; NOMO, nodal modulator; TM, transmembrane domain.

As a first step toward a better structural understanding of NOMO1, we set out to determine the overall architecture of the molecule. NOMO1-FLAG was purified from Expi293F cells, and the sample was analyzed by negative-stain EM. Two-dimensional class averages were generated using RELION from 7000 particles. The top two-dimensional class averages from the collected dataset feature a flexible, extended rod of about 30 nm (Fig. 8*A*). A three-dimensional model obtained from these data is 27 nm in length, similar to the ER diameter measured by EM under NOMO overexpression (Figs. 6*E* and 8*B*).

To determine if a NOMO monomer alone would be similar in length as suggested by the SEC-MALS data, a negative-stain structure was also determined for NOMO^LD^-FLAG. NOMO^LD^-FLAG was purified from Expi293F cells and visualized by negative-stain EM. The two-dimensional classifications were generated using RELION from 9000 particles and again a flexible and somewhat thinner rod-shaped molecule compared with the full-length protein, consistent with the monomeric nature of this NOMO^LD^ construct. Interestingly, the class averages feature a “beads on a string” morphology with eight discernable globular segments (Fig. 8*C*), probably accounting for Ig-like domains, given the structural homology to proteins with Ig-like folds. The obtained three-dimensional model is about 24 nm in length. Because the ends of the particles appear to be blurry, we cannot exclude the possibility that additional Ig folds exist at the ends that we were not able to resolve because of structural flexibility, especially considering the extensive beta sheet–rich LD of NOMO1 (Fig. S1*B*), which could potentially give rise to more than eight Ig folds. To try to optimize the negative-stain models, automated particle picking with crYOLO (44) was performed, but both crYOLO and manual picking led to similar two-dimensional class averages and three-dimensional reconstructions produced by RELION (Fig. S3, *A* and *B*). In conclusion, NOMO1 is a flexible, rod-shaped protein featuring a “beads on a string” arrangement of at least eight consecutive domains, several or all of which may represent Ig-like folds.

### Establishing a relationship between molecular dimensions of NOMO1 and intermembrane spacing

**Figure 9:**
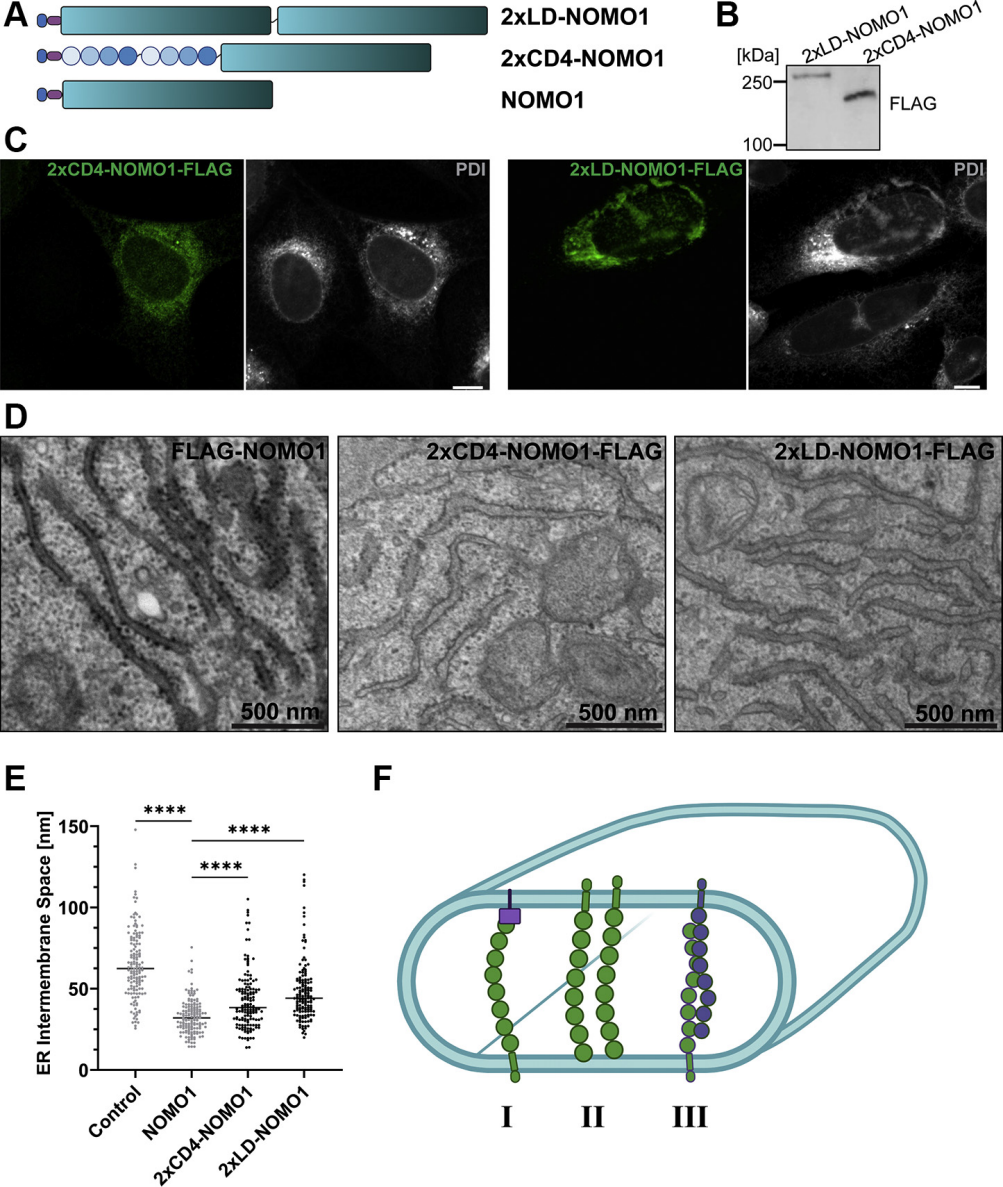
**Extended NOMO constructs increase the ER intermembrane space.***A*, design of the two extended NOMO constructs (see the text for details). *B*, detection of the extended constructs *via* SDS-PAGE/immunoblotting using a FLAG antibody. *C*, validation of ER localization by immunofluorescence. PDI is used as the ER marker. All scale bars are 10 μm. *D*, representative EM images of HeLa cells expressing the respective construct. *E*, quantification of the ER intermembrane space for each transfected construct compared with NOMO1-FLAG and control cells. ∗∗∗∗*p* < 0.0001. *F*, models for membrane spacing by NOMO1. I, the *purple block* represents an unknown/speculative interaction partner. II, NOMO interacts with the membrane. III, NOMO forms an antiparallel dimer. ER, endoplasmic reticulum; NOMO1, nodal modulator 1; PDI, protein disulfide isomerase.

To further test our hypothesis that NOMO contributes to membrane spacing, we engineered two longer NOMO variants (Fig. 9*A*) and asked whether the intermembrane distance can be increased through insertion of additional Ig folds between the LD and TM domains of NOMO1. The first construct, designated 2xLD-NOMO1-FLAG, features a duplicated NOMO LD, which produces a 268-kDa protein with an estimated luminal length of 48 nm. The second construct, 2xCD4-NOMO1-FLAG, features two consecutive copies of four Ig-fold domains of the human T-cell surface glycoprotein CD4 (45). This approach was previously used to elongate a single-chain trimer ectodomain involved in T-cell triggering to observe an increase in the intermembrane space of a cell-to-cell interface (45). The resulting eight additional Ig folds have a length of ∼20 to 24 nm. Consequently, 2xCD4-NOMO1 produces a 221-kDa protein with an estimated LD length of 44 to 48 nm.

First, the expression and localization of the constructs were validated *via* immunoblotting and immunofluorescence (Fig. 9, *B* and *C*). These constructs were then transfected into HeLa cells, which were processed for thin-section EM. The ER lumen did appear wider in the EM images (Fig. 9*D*). Indeed, a quantification of the intermembrane distance revealed a significant increase for both extended constructs (Fig. 9*E*). 2xCD4-NOMO1-FLAG increased the average intermembrane distance to 41 nm, a 1.2-fold increase relative to the 33-nm spacing imposed by NOMO1 alone, while 2xLD-NOMO1 resulted in an average intermembrane distance of 47 nm, a 1.4-fold increase in the intermembrane space. Thus, a correlation exists between the molecular dimensions of NOMO1 and its extended variants and the ER intermembrane space.

## Discussion

In this study, we performed an unbiased proteomics-based experiment to identify abundant, ER-luminal proteins that could serve a function as architectural components of the ER. We identified NOMO1 as an abundant ER constituent of unknown function (Fig. 1), motivating our functional characterization in the context of ER morphology. Notably, NOMO1 and NOMO2 have previously been observed in ER proteomes (22, 46), but remained uncharacterized. NOMO was first described in zebrafish as a nodal signaling regulator (30). The nodal signaling pathway is an embryonic developmental signaling pathway important for cellular differentiation (47). The ectopic expression of NOMO and NCLN, a NOMO-binding partner, leads to cycloptic embryos in zebrafish (30). TMEM147 was later found to form a complex with NOMO and NCLN (37). NCLN and TMEM147 were recently shown to associate with Sec61 and linked to a role in membrane protein biogenesis (48). However, the solved structure of this complex did not contain NOMO1, leaving the molecular function of NOMO unresolved.

Our morphological characterization of NOMO-depleted cells revealed a drastic rearrangement of the ER network, creating vacuole-like holes in the ER network (Fig. 2*A*). This phenotype was rescued by overexpression of Atl2 and Climp63. This suggests that the hole phenotype is likely due to an architectural problem because Atl2 and Climp63 provide structural support to the ER, connecting NOMO to the network of known ER-shaping proteins.

Ultrastructural analysis of the holes that arise upon NOMO depletion reveal an enrichment of lysosome-like, electron-dense structures (Fig. 4*A*). Consistently, LC3-II and p62 levels increased, consistent with an increase of autophagic flux or partial perturbation of autophagy (Fig. 5, *C* and *D*). Of note, NCLN or TMEM147 depletion did not provoke an increase of LC3-II (Fig. 5*B*), neither did we observe rearrangements of the ER network in this experimental context (Fig. S4). Thus, NOMO1 can likely function independently of the NCLN–TMEM147 complex. We did not observe an induction of the UPR in NOMO-depleted cells (Fig. 5*C*), arguing against a critical function for membrane protein biogenesis. However, we cannot formally exclude subtle folding defects that would not amount to a UPR induction. Another possibility is that NOMO could additionally serve as a sheet anchor for the NCLN–TMEM147–Sec61 complex to recruit the process of biogenesis of certain polytopic proteins to flat regions of the membrane.

Regardless, our observation of changes in autophagic/lysosomal route upon NOMO depletion stresses the relationship of form and function of the ER. Besides imposing a distinct shape on subcompartments of the ER, ER-shaping proteins are important for defining distinct identities of these compartments. It is interesting to note that while Atl2 depletion results in LAMP1-positive compartments but not an LC3-II or p62 increase, Climp63 depletion does not provoke enlarged lysosomes but does result in an LC3-II and p62 increase. On the other hand, NOMO depletion causes both a robust increase in LC3 levels and LAMP1-positive compartments (Fig. 5). While ATLs, Rtn3, and Lunapark have been previously linked to autophagy (49, 50, 51, 52), the connection of NOMO1 and Climp63 to the autophagic/lysosomal route is to our knowledge underexplored and warrants closer scrutiny in the future.

Overexpression of NOMO1 resulted in a restriction of the ER luminal diameter to about 30 nm (Fig. 6*E*). This was particularly interesting because Climp63 depletion results in a decrease of the ER lumen to 30 nm (16, 19), implying that NOMO may be among the remaining sheet-shaping proteins responsible for this smaller diameter of 30 nm. Our structural analysis revealed that NOMO1 is an extended, flexible rod of about 27-nm length, which is similar to the diameter that NOMO1 overexpression imposes on the ER lumen. We speculate that the flexibility of NOMO1 revealed by the negative-stain particles may be a structural feature to prevent an overly rigid property of ER sheets. Climp63 had been proposed to be a stable coiled coil dimer (15). More recently, calumenin-1 was recently shown to regulate Climp63’s distribution across ER sheets (19), allowing the ER to adapt and respond to physiological demands that require different distributions of sheets *versus* tubules.

The NOMO^LD^ model revealed that NOMO1 features at least eight discernable domains that are arranged as “beads-on-a-string” domains (Fig. 8*B*), reminiscent of the POM152 structure composed of nine Ig folds (53). POM152 is a nuclear pore protein with a small cytosolic domain, single TM domain, and large LD of 1144 residues, which also was found to have homology to bacterial proteins with Ig-like folds (53). Considering that Ig domains can have high structural similarity without significant sequence homology (54), and predicted structural similarity to Ig-fold proteins, our interpretation is that each of these segments correspond to one Ig fold domain, consistent with the secondary structure prediction showing a high beta sheet content for nearly the entire sequence of NOMO1 (Fig. S1*B*). It should be noted that based on both the primary structure dimensions and secondary structure predictions (Fig. S1*B*), it is possible that the LD contains more than eight Ig folds that we cannot directly observe because of structural flexibility. Interestingly, structurally related pilin proteins in bacteria featuring extensive Ig fold regions can dissipate mechanical forces by acting as molecular shock absorbers (55). Thus, it will be interesting to test if NOMO fulfills a similar function in the ER and explore possible links to the cytoskeleton.

Importantly, we were able to show that tuning the length of NOMO by insertion of defined Ig-fold segments between the TM and LD domains resulted in a correlative increase in the intermembrane distance in the ER (Fig. 9, *C* and *D*). This result supports the idea that NOMO1 contributes to membrane spacing along with established players such as Climp63. Some degree of functional overlap might exist and would be consistent with our observation that Climp63 overexpression can rescue the NOMO depletion phenotype (Fig. S2*A*).

How can we reconcile the dimensions of NOMO1 with our proposed role as a sheet-shaping protein? We consider three models to relate the dimensions of the NOMO1 rod to the intermembrane spacing observed upon NOMO1 overexpression. First, another, yet unidentified, protein interacts with the distal luminal end of NOMO1 at the opposite membrane (Fig. 9*F*, I). Second, the distal luminal end of the rod-shaped molecule interacts with the membrane itself (Fig. 9*F*, II). Third, NOMO1 forms antiparallel dimers or oligomers of weak affinity (Fig. 9*F*, III) such that these interactions are not necessarily captured by SEC-MALS analysis. Indeed, a number of distinct oligomeric states of Climp63 were recently observed by analytical ultracentrifugation (56). If NOMO or Climp63 require an interaction partner to induce their sheet-shaping functions (Fig. 9*F*, I), then overexpressing NOMO or Climp63 would not necessarily cause a striking constriction of the ER intermembrane spacing as the quantity of the interaction partner could be a limiting component. The direct membrane interaction model (Fig. 9*F*, II) or antiparallel oligomers model (Fig. 9*F*, III) do not rely on the presence of an interaction partner and could more readily explain the observed correlation between NOMO1 expression levels and the ER intermembrane space. Clearly, additional experiments will be required to test these models in the future.

In conclusion, we identified a critical role for NOMO1 in sustaining the morphology of the ER. We propose a dynamic model where both the molecules responsible for membrane spacing and the interactions between them or their interaction partners are highly dynamic. This could be achieved by the inherent flexibility of membrane-spacing proteins as exemplified by NOMO1, as well as low- to moderate-affinity interactions with binding partners at the opposite membrane. In line with this model, homotypic Climp63 interactions appear to be weak (56). A dynamic model relying both on avidity of multiple weak interactions and inherent flexibility would ensure that ER spacers do not form an impediment for the secretion of bulky cargo (*e.g.*, procollagen with 300- to 450-nm length (57)) and allow for rapid adjustments of the ER morphology in response to mechanical challenge or physiological demand.

## Experimental procedures

### Tissue culture and stable cell line generation

U2OS and HeLa cells from the American Type Culture Collection were maintained at 37 °C and 5% CO_2_ and regularly passaged in Dulbecco's modified Eagle's medium supplemented with 10% (vol/vol) fetal bovine serum (Gibco) and 1% (vol/vol) Penicillin/Streptomycin (Gibco). Expi293F cells were maintained at 37 °C and 8% CO_2_ in Expi293F expression media and passaged to maintain a density of less than eight million cells per milliliter.

U2OS and HeLa cells were transfected with plasmids using X-tremeGene 9 or FuGENE 6, according to the manufacturer’s protocol, 24 h before fixing with 4% paraformaldehyde in PBS. For rescue assays, U2OS cells were cotransfected with the DNA plasmid and siRNA using Lipofectamine 2000 for 48 h.

For siRNA transfections, RNAi Lipofectamine was used to transfect U2OS and HeLa cells. siRNA was used at a final sample concentration of 50 nM. A double-dose protocol was followed for NOMO and Climp63 depletion, where the cells were transfected with siRNA on the first day, transfected again with siRNA 24 h later, and fixed with 4% (vol/vol) paraformaldehyde in PBS 48 h after the second transfection.

NOMO and Climp63 were depleted with ON-TARGETplus SmartPools from Dharmacon. Atlastin2 was depleted using the siRNA as in (58).

### APEX2 and MS

ER-APEX2 was transfected into 2 × 10 cm plates of HeLa cells using X-tremeGene 9 and expressed overnight. 16 to 18 h later, cells were incubated with 500 μM biotin-phenol for 30 min and then treated with 1 mM hydrogen peroxide, from a freshly diluted 100 mM stock, for 1 min before being quenched with 2× quenching buffer. The quenching buffer (2×) contained 50 mg Trolox and 80 mg sodium ascorbate in 20 ml of PBS. Cells were rinsed with 1× quenching buffer twice and once with PBS. One control plate was not treated with hydrogen peroxide but was still rinsed with 1× quenching buffer and PBS. Trypsin (0.05%) was then added to the cells for collection into a microfuge tube. Cell samples were spun down at 800*g* for 3 min at 4 °C, rinsed once with PBS, spun down again at 0.8*g* for 3 min at 4 °C, and then lysed in an SDS buffer, before quantifying proteins concentrated with a BCA Assay (Thermo Fisher). The original protocol can be found in (26). Equal amounts of lysate samples were incubated with 30 μl streptavidin resin for 3 h. The beads were washed three times and then eluted using 2× Laemmli Sample Buffer (Bio-Rad). The elution was subjected to SDS-PAGE. The lane was then excised into 2 to 3 bands and submitted for MS analysis.

MS samples were analyzed after tryptic digestion by the Mass Spectrometry (MS) & Proteomics Resource of the W.M. Keck Foundation Biotechnology Resource Laboratory located at the Yale School of Medicine and using an LTQ Orbitrap XL (Thermo Scientific). Mascot, version 2.6.0, was used as the search engine using the SwissProt_2017_01.fasta tax: *Homo sapiens* database. 41,791 entries were searched in MS/MS Ion search mode. The maximum number of missed cleavages was set to 2. Oxidation (M) and Carbamidomethyl (C) were the variable modifications considered. The peptide mass tolerance was 10 ppm. The fragment mass tolerance was 0.02 Da. The significance threshold was *p* < 0.05. The false discovery rate for peptide matches above the identity threshold is 3.49%.

### Immunofluorescence

Imaged cells were fixed in 4% (vol/vol) paraformaldehyde/PBS for 15 min and permeabilized with 0.1% Triton X-100/PBS for 10 min before blocking with 4% (wt/vol) BSA/PBS for another 10 min. Samples were then incubated with primary antibodies diluted to 1:500 in 4% BSA/PBS and secondary antibodies diluted to 1:700 in 4% BSA/PBS for 1 h each. Samples were rinsed three times with PBS between and after antibody incubations and mounted onto slides using Fluoromount-G (SouthernBiotech).

For samples where the LAMP1 antibody was used, a gentle permeabilization method was followed. After being fixed in 4% (vol/vol) paraformaldehyde/PBS for 10 min, cells were gently permeabilized with a solution of 0.05% (wt/vol) saponin and 0.05% (vol/vol) NP-40/PBS for 3 min. The cells were then rinsed with 0.05% saponin/PBS and incubated with primary and secondary antibodies, respectively, diluted in 0.05% saponin and 1% BSA/PBS. Samples were then rinsed with PBS and mounted onto slides using Fluoromount-G.

Quantification on ImageJ of band intensity was done by converting the immunoblot image to an 8-bit image and creating a binary image to highlight and convert the relevant bands to pixels. The pixels were then measured with the “Analyze Particles” tool. “Show: Results” was selected to label the bands in a binary image with relevant pixel quantification.

### Rescue assays

The rescue assays shown in Figures 2 and 4 were done as follows. About 60,000 U2OS cells were plated on a coverslip in a 12-well dish 24 h before transfection. For each sample, 50 nM of the respective siRNA was incubated with 500 ng of the respective DNA in 50 μl of Opti-MEM for 5 min. At the same time, 2 μl of Lipofectamine 2000 (Invitrogen) was diluted into 50 μl of Opti-MEM. After 5 min, the diluted Lipofectamine was added to the siRNA/DNA and mixed gently. The mixture was left to incubate for 20 min at room temperature (RT). Each 12-well was replaced with 900 μl of fresh media. The siRNA/DNA/Lipofectamine mixture was added to the well gently. The media was replaced 12 to 16 h later. The cells were fixed 48 h after the initial transfection. The samples were then processed for immunofluorescence as described above and imaged on a Zeiss Axiovert Observer D1 with a 63×/1.4 oil immersion lens and an AxioCam MRm camera. A minimum of 100 transfected cells were imaged for each sample for each replicate. Cells with two or more holes of 3 μm or greater were considered to have the hole-depletion phenotype and considered not rescued. The four relevant plasmids, NOMOr-FLAG, Climp63-FLAG, Alt2-FLAG, and Atl2K107A-FLAG, were blinded by a fellow laboratory member before transfections occurred. This experiment was repeated four times for each siRNA and plasmid combination.

### Confocal imaging

Immunofluorescent images were acquired on a Zeiss LSM 880 with Airyscan capabilities using a 63× objective with a 1.4 numerical aperture. The pinhole is 0.8 μm per section. ImageJ was used to crop the images if necessary. The scale bar was also inserted using ImageJ as the embedded metadata allowed for ImageJ to add the appropriately sized scale bar.

### Antibodies

The following antibodies were used: Protein disulfide isomerase (PDI), Abcam, ab2792; BiP, Abcam, ab21685; Actin, Abcam, ab8226; Alpha-Tubulin, Sigma, T5168; LAMP1, BioLegend, 328602; Calnexin, Abcam, ab75802; FLAG, Sigma, F1804; LC3, Novus, NB100-2331; SQSTMI/p62, Abcam, 207305.

### TEM

The Center for Cellular and Molecular Imaging Electron Microscopy Facility at the Yale School of Medicine prepared the samples. Cells were fixed in 2.5% (vol/vol) glutaraldehyde in 0.1 M sodium cacodylate buffer plus 2% (wt/vol) sucrose, pH 7.4, for 30 min at RT and 30 min at 4 °C. After rinsing, cells were scraped in 1% (wt/vol) gelatin and centrifuged in a 2% (wt/vol) agar solution. Chilled cell blocks were processed with osmium and thiocarbohydrazide-osmium liganding. Samples were incubated overnight at 60 °C for polymerization. The blocks were then cut into 60-nm sections using a Leica UltraCut UC7 and stained with 2% (wt/vol) uranyl acetate and lead citrate on Formvar/carbon-coated grids. Samples were imaged using a FEI Tecnai BioTwin at 80 kV, equipped with a Morada CCD and iTEM (Olympus) software for image acquisition.

### TEM image quantification

The intermembrane spaces quantified in the article were quantified from EM images. A minimum of 15 cells were quantified for each sample. When a cell was imaged, the image acquisition software embedded the appropriate scale bar. The images were opened on a 20-inch monitor and analyzed with ImageJ. The embedded scale bar was used to set the scale bar in ImageJ. The line tool was then used to manually measure the intermembrane distance of the visible ER cross-sections. A measurement was taken for every inch of visible ER cross-section. If a total of more than 150 measurements were measured from a total of 15 cells or more, the measurements were randomized using the Excel random number function and the first 150 measurements were used for quantification.

### Cloning, expression, and purification of NOMO constructs

The following constructs were cloned using Gibson assembly from Dharmacon plasmids containing the original gene into a pcDNA3.1+ vector with a C-terminal FLAG tag: NOMO1-FLAG, FLAG-CLIMP63, ATL2-FLAG. NOMO^LD^-FLAG was subcloned from NOMO1-FLAG using Gibson assembly to include only residues 1 to 1160. FLAG-NOMO1 was cloned using the Dharmacon cDNA to PCR residues 33 to 1226 into a pcDNA3.1+ vector with an N-terminal MHC I signal sequence followed by a FLAG tag. 2xLD-NOMO was cloned using Gibson assembly to insert a copy of the LD, residues 22 to 1160, between the LD and TM domain, with a GSGS linker between the two LDs. 2xCD4-NOMO1-FLAG was cloned by synthesizing the 2xCD4 insert (Integrated DNA technologies, Inc). The 2xCD4 insert corresponds to two copies of residues 1 to 363 of CD4 with a GSGS linker between the two copies, as well as a GSGS linker before and after the 2xCD4 insert. Gibson assembly was then used to insert the 2xCD4 fragment in between the NOMO LD and TM domain.

Expi293F cells were transfected with the construct of interest using the ExpiFectamine 293 Transfection Kit (Gibco) following the manufacturer’s protocol for a 50-ml culture. Cells were harvested 72 h after transfection and frozen at −80 °C. Cell pellets were thawed on ice and lysed in buffer A (50 mM MES, 100 mM NaCl, 50 mM KCl, 5 mM CaCl_2_, pH 6.0), 5% glycerol, and 1% DDM for 1 h at 4 °C. Afterward, samples were spun for 30 min at 20,000*g* at 4 °C. The supernatant was incubated with anti-FLAG M2 beads (Sigma) overnight and then loaded into a gravity column for washing before incubating with the elution buffer containing 5 μM FLAG peptide for 30 min. The elution was then concentrated to 0.5 ml and subjected to SEC in an S200 or S75 column (GE Healthcare). DDM (0.05%) was added to buffer A for full-length NOMO and 0.005% DDM for NOMO^LD^, 2XFLAG-MBP-CYT, and 2XFLAG-MBP.

### SEC-MALS

Multiangle laser light-scattering experiments were performed at RT in a 50 mM MES, pH (6.0), 150 mM KCl, 5 mM MgCl_2_, 5 mM CaCl_2_, 2% (vol/vol) glycerol, and 0.05% (wt/vol) DDM buffer for NOMO1-FLAG and MBP-TM-CYT. NOMO^LD^ was run in the same buffer but at 0.005% DDM. Light-scattering data were collected using a DAWN Heleos-II spectrometer (Wyatt Technology) coupled to an Optilab T-rEX (Wyatt Technologies) interferometric refractometer. Samples (500 μl) were injected and run over a Superose 6 Increase or Superdex 200 Increase 10/300 GL column (GE Healthcare) at a flow rate of 0.5 ml/min. Light scattering (690 nm laser), UV absorbance (280 nm), and refractive index were recorded simultaneously during the SEC run. Before sample runs, the system was calibrated and normalized using the isotropic protein standard, monomeric bovine serum albumin. Data were processed in ASTRA software. A protein conjugate analysis was done to correct for detergent contributions.

### Single-particle EM

Purified NOMO1-FLAG or NOMO^LD^-FLAG (3.5 μl) was negatively stained using 2% uranyl acetate solution on carbon film and 400 mesh copper grids that were glow discharged. Grids were imaged on a FEI Talos L120C Electron Microscope (Thermo Fisher Scientific) at 120 kV. Micrographs were captured at a magnification of 73,000×. 82 and 61 micrographs were taken for NOMO and NOMO^LD^, respectively. TIFF files were cropped to 4096 × 4096 pixels and converted to MRC format using the EMAN2 v2.3 (59) *eproc2d* program. Two-dimensional classifications and three-dimensional reconstructions were produced using RELION, v3.08 (60), with manually picked particles. Contrast transfer function estimation was performed using CTFFIND 4.1 with box sizes of 512 and 352 pixels for NOMO and NOMO^LD^, respectively. Particles were extracted and then downscaled 4-fold for two-dimensional class averages. Selected two-dimensional classes used for three-dimensional reconstruction are shown in Figure 8. Final three-dimensional volumes were generated by applying masks generated from initial models and auto-refinement in RELION. Manually picked particles were used to train crYOLO (44) for automated particle picking.

### Baf A assay

U2OS cell samples were treated with 20 μM Bafilomycin A (Sigma) for 4 h. The Baf A stock was made at 1 mM in DMSO. Cell samples were then trypsinized, spun down for 3 min at 800*g*, and lysed in a 1% SDS buffer. Benzonase (0.25 μl) was added to each sample. The sample was left to incubate at RT for 10 min before heating at 60 °C for 5 min before performing a BCA assay to determine protein concentration. Ten microgram of each sample was loaded onto an Any kD precast gel (Bio-Rad) for optimal resolution followed by immunoblotting.

## Data availability

The HeLa cell ER-APEX2 Mass Spectrometry Dataset has been made available at https://zenodo.org/record/4914811#.YL_13_lKhyw. All remaining data are contained within the article and supplemental information.

## Supporting information

This article contains supporting information.

## Conflicts of interest

The authors declare that they have no conflicts of interest with the contents of this article.
