# Supplementary material for: Nodal modulator (NOMO) is required to sustain endoplasmic reticulum morphology
Source: J Biol Chem. 2021 Jul 3;297(2):100937. doi: 10.1016/j.jbc.2021.100937 (PMC8327139; doi:10.1016/j.jbc.2021.100937)
Supplement: Supplemental Figures S1–S4 and Table S1 [file mmc1.docx]

**Nodal modulator (NOMO) is required to sustain endoplasmic reticulum morphology­**

Catherine Amaya, Christopher JF Cameron*, Swapnil C. Devarkar*, Sebastian JH Seager, Mark B Gerstein, Yong Xiong, Christian Schlieker

**Supporting Information**

List of material:

Table S1

Figure S1

Figure S2

Figure S3

Figure S4


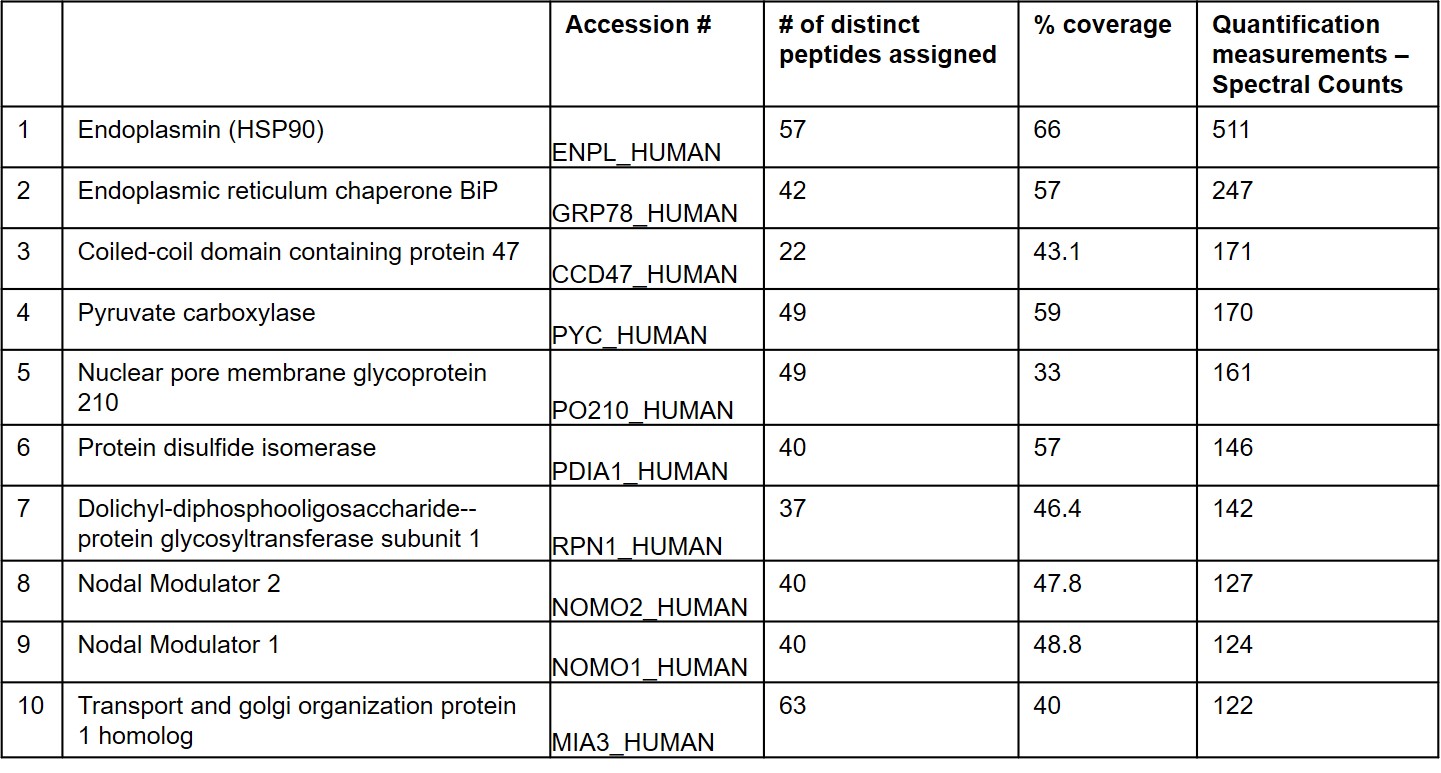


Table S1. Mass spectrometry information of the top ten abundant proteins of the ER-APEX2 mass spectrometry analysis.


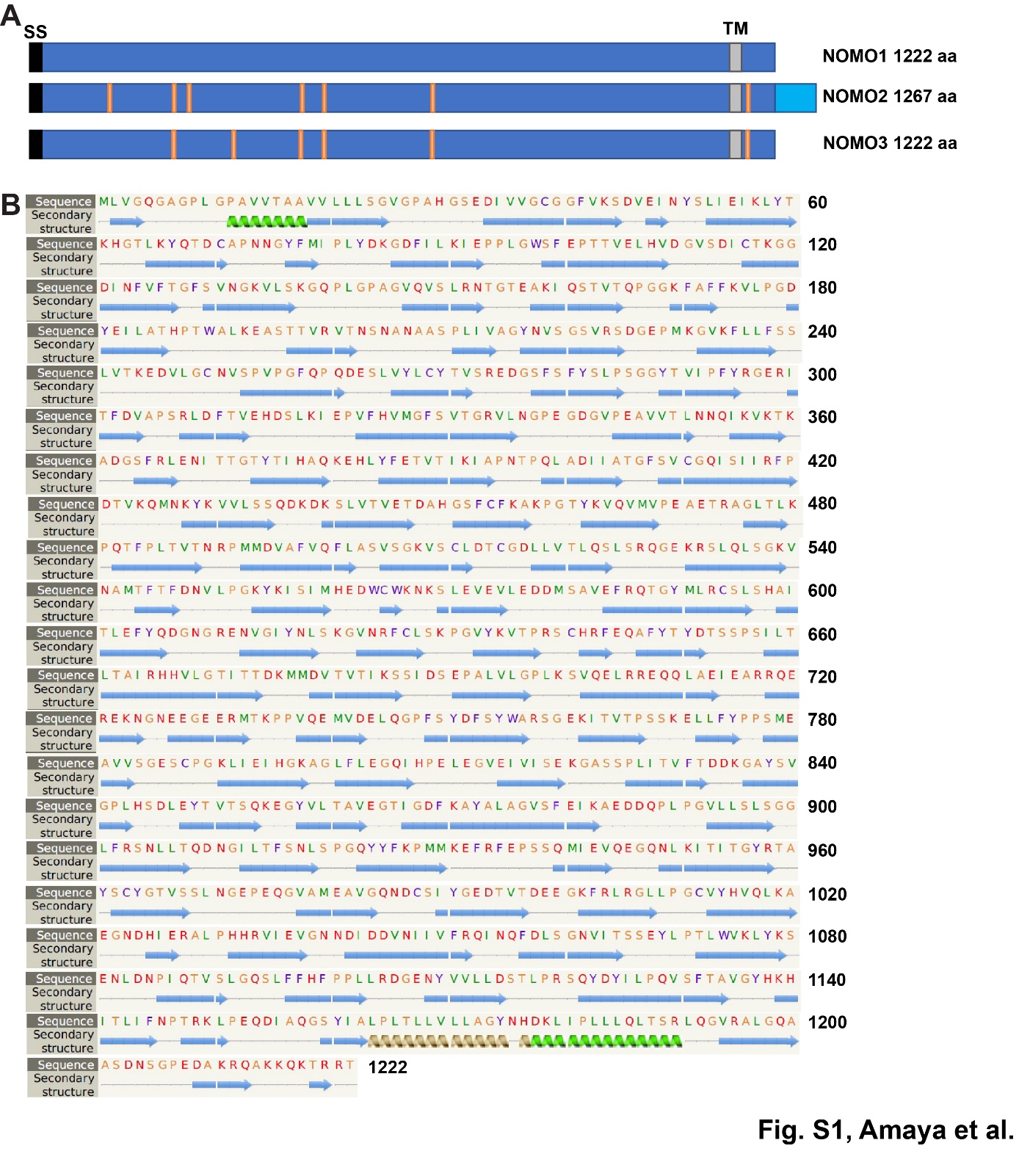


Figure S1. Domain organization of NOMO1. A. NOMO1, NOMO2, and NOMO3 isoforms. Orange denotes single amino acid differences between isoforms. The extended cytosolic tail of NOMO2 is shown in light blue. SS= signal sequence, TM= transmembrane domain. B. NOMO1 secondary structure prediction from Phyre2.


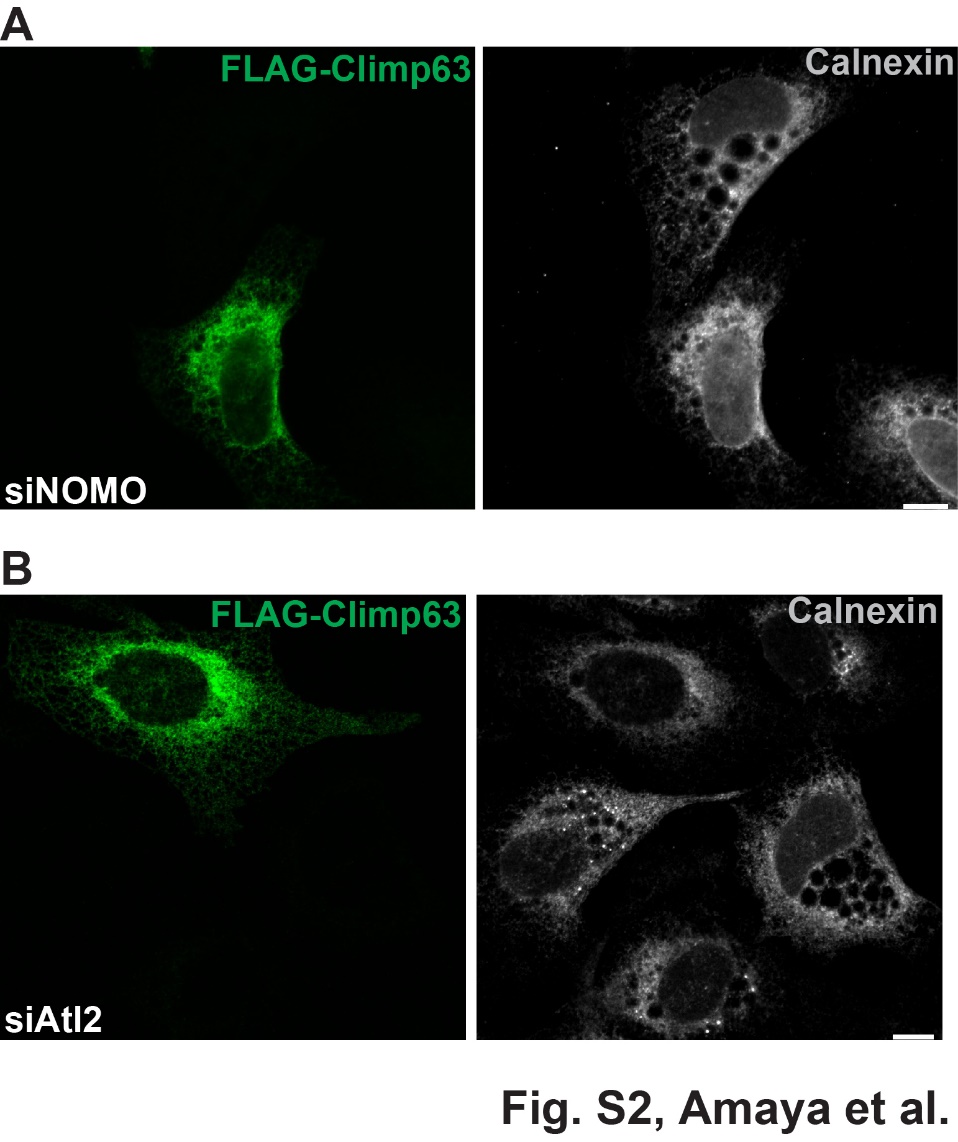


Figure S2. Climp63 rescues siNOMO and siAtl2 phenotypes. A. Representative confocal image of FLAG-Climp63 rescuing the siNOMO phenotype, calnexin was used as ER marker. B. Representative confocal image of FLAG-Climp63 rescuing the siAtl2 phenotype, calnexin was used as ER marker. Scale bars are 10 µm.


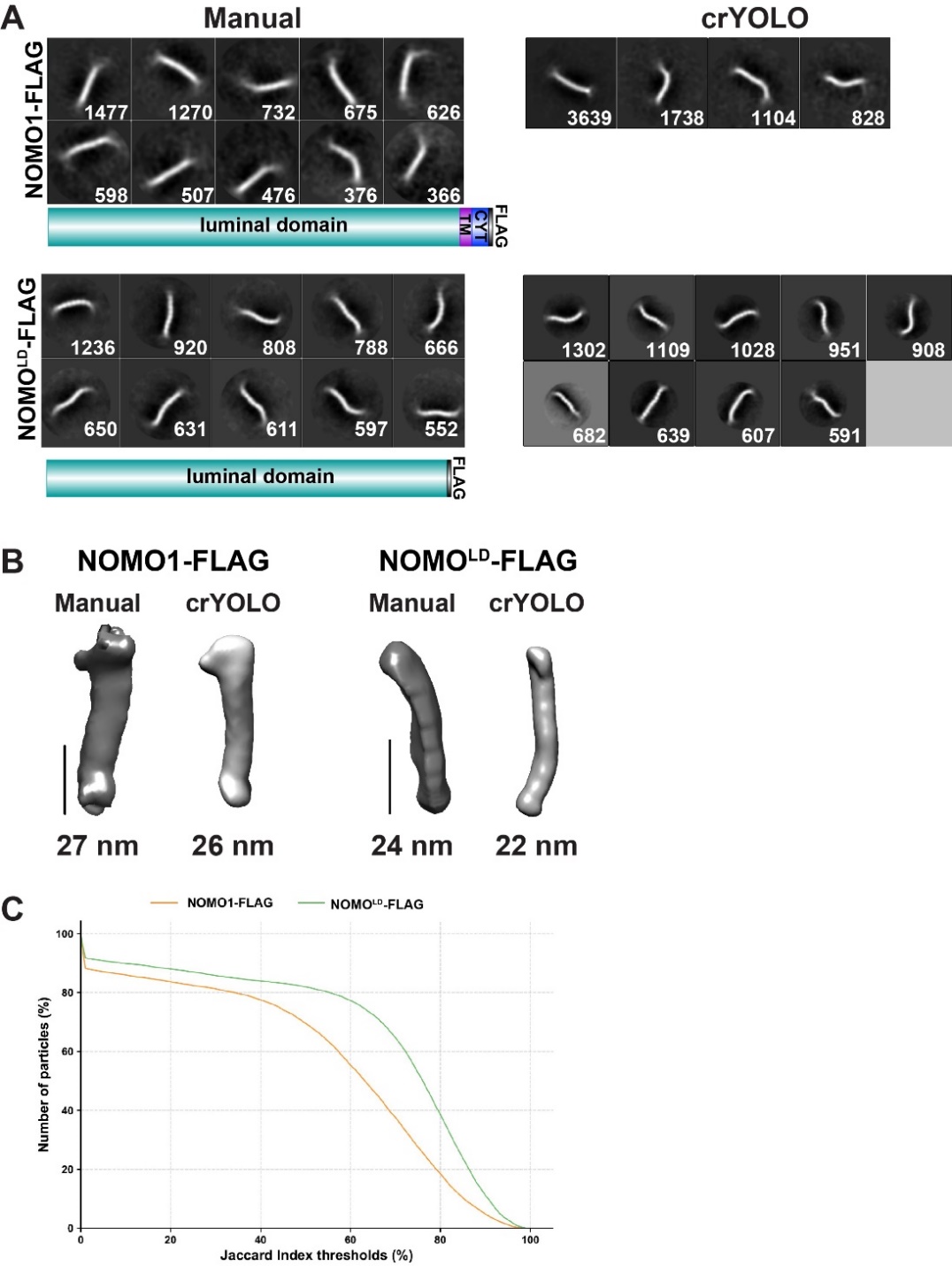


Figure S3. A. Selected RELION 2D class averages for manual and crYOLO picked particle sets. The manual 2D classes were previously presented in Figure 8. The images are reused here to ensure a direct comparison between the manual and crYOLO class averages. Manual picking resulted in 9,041 and 10,177 particles for NOMO1-FLAG and NOMO^LD^-FLAG, respectively. Mask diameters are 40 nm and 30 nm for NOMO1-FLAG and NOMO^LD^-FLAG, respectively. crYOLO picking resulted in 10,347 and 13,120 particles for NOMO1-FLAG and NOMO^LD^-FLAG, respectively**.** Mask diameters are 42 nm and 23 nm for NOMO1-FLAG and NOMO^LD^-FLAG, respectively. B. Refined 3D models produced by RELION using 2D class averages. Model length is below each reconstruction**.**  C. Lines represent the overlap between manual and crYOLO particle image sets at various Jaccard Index thresholds. Both methods selected similar particles, where ~60% or ~80% (orange and green lines, respectively) of particles overlap with a Jaccard Index of 60% using a maximum bipartite matching algorithm.


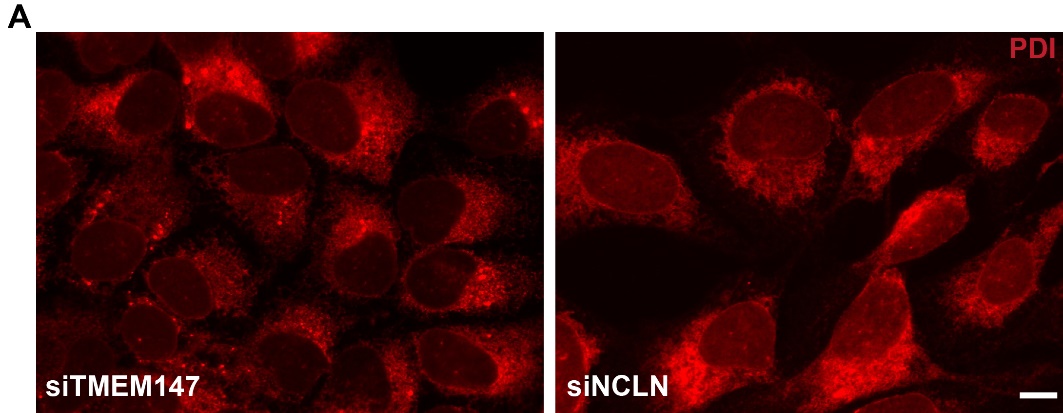


Figure S4. Depletion of NOMO interaction partners do not disrupt ER morphology. A. U2OS cells were treated with the denoted siRNAs for 48 hrs and imaged via immunofluorescence. Scale bar is 10 µm.
